# Supplementary material for: The tail domain of the plant kinesin-12 POK2 is a versatile interaction hub
Source: J Cell Sci. 2025 Oct 10;138(19):jcs263785. doi: 10.1242/jcs.263785 (PMC12516131; doi:10.1242/jcs.263785)
Supplement: Supplementary information [file joces-138-263785-s1.pdf]

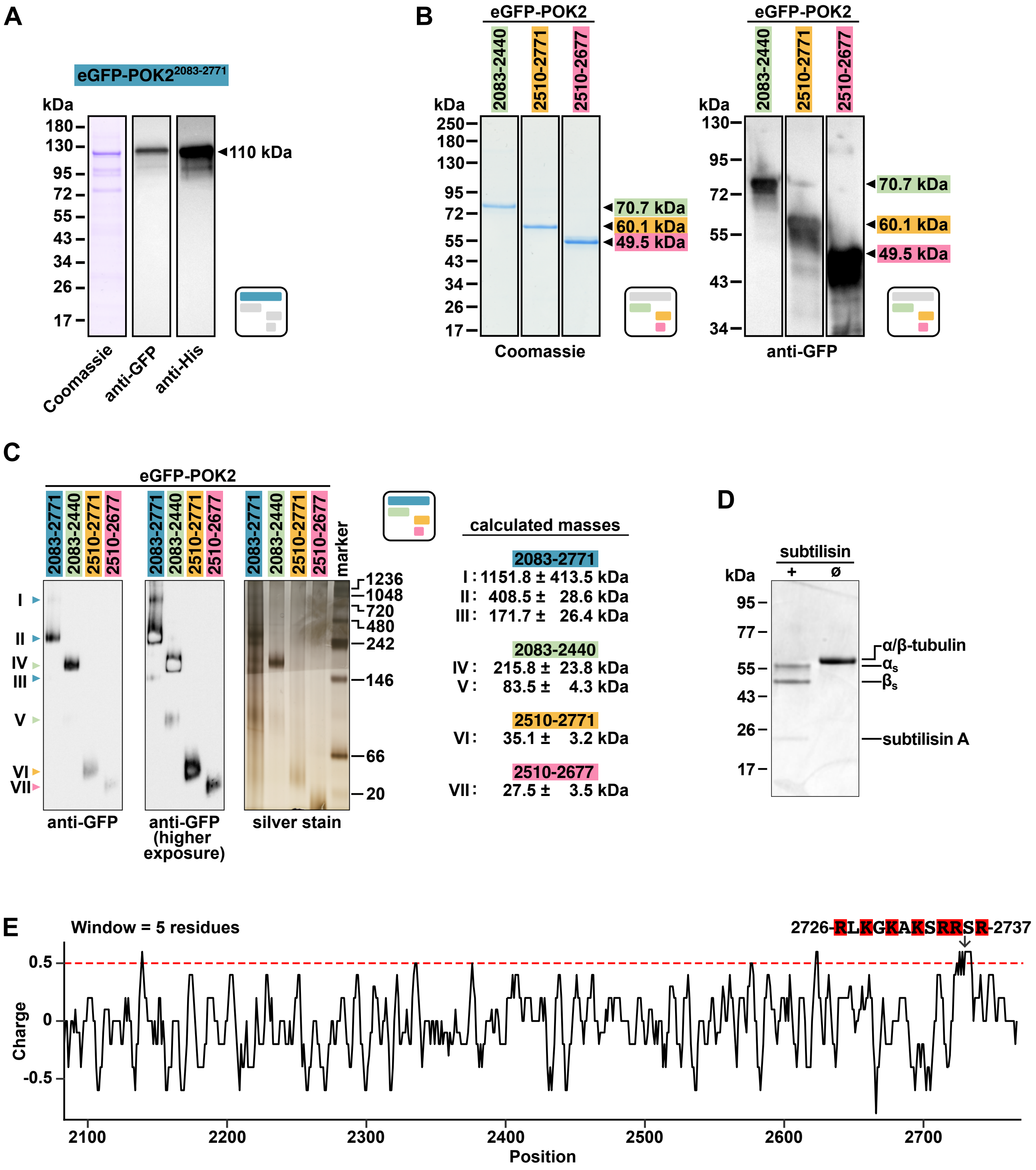

Fig. S1.

(A) Images of Coomassie stained and anti-GFP and anti-His western blotted SDS-PAGE gels of purified eGFP-POK2$^{2083\text{-}2771}$-8xHis. While there are some degradation products, the major band in the Coomassie stain corresponds to the bands in the western blots and is roughly the predicted molecular weight, running slightly higher (arrowhead).

(B) Images of Coomassie stained and anti-GFP western blotted SDS-PAGE gels of purified shorter eGFP- and -8xHis tagged POK2 constructs which also run slightly higher than predicted. Arrowheads indicate band expected to correspond to desired recombinant protein and the predicted molecular weight.

(C) Images of anti-GFP western blotted and silver stained blue native PAGE gels of purified POK2 tail constructs. Band sizes deviate from predicted sizes, as is common in native PAGE. Roman numbers indicate bands for which their apparent molecular weight have been calculated based on the marker retention during the gel run, with means ± standard deviation listed to the right of the gels.

(D) Images of Coomassie stained SDS-PAGE gel of 20% biotinylated double-stabilised microtubules treated with subtilisin A (+) and untreated control (ø).

(E) Charge prediction along the amino acid sequence of the C terminus of POK2 (EMBOSS Charge) using a window size of 5 residues. Highlighted is a region of amino acids with a predicted charge above +0.5.

Square icons follows color scheme and layout of POK2 recombinant proteins in Fig. 1A, placed here for easy reference.

Journal of Cell Science • Supplementary information

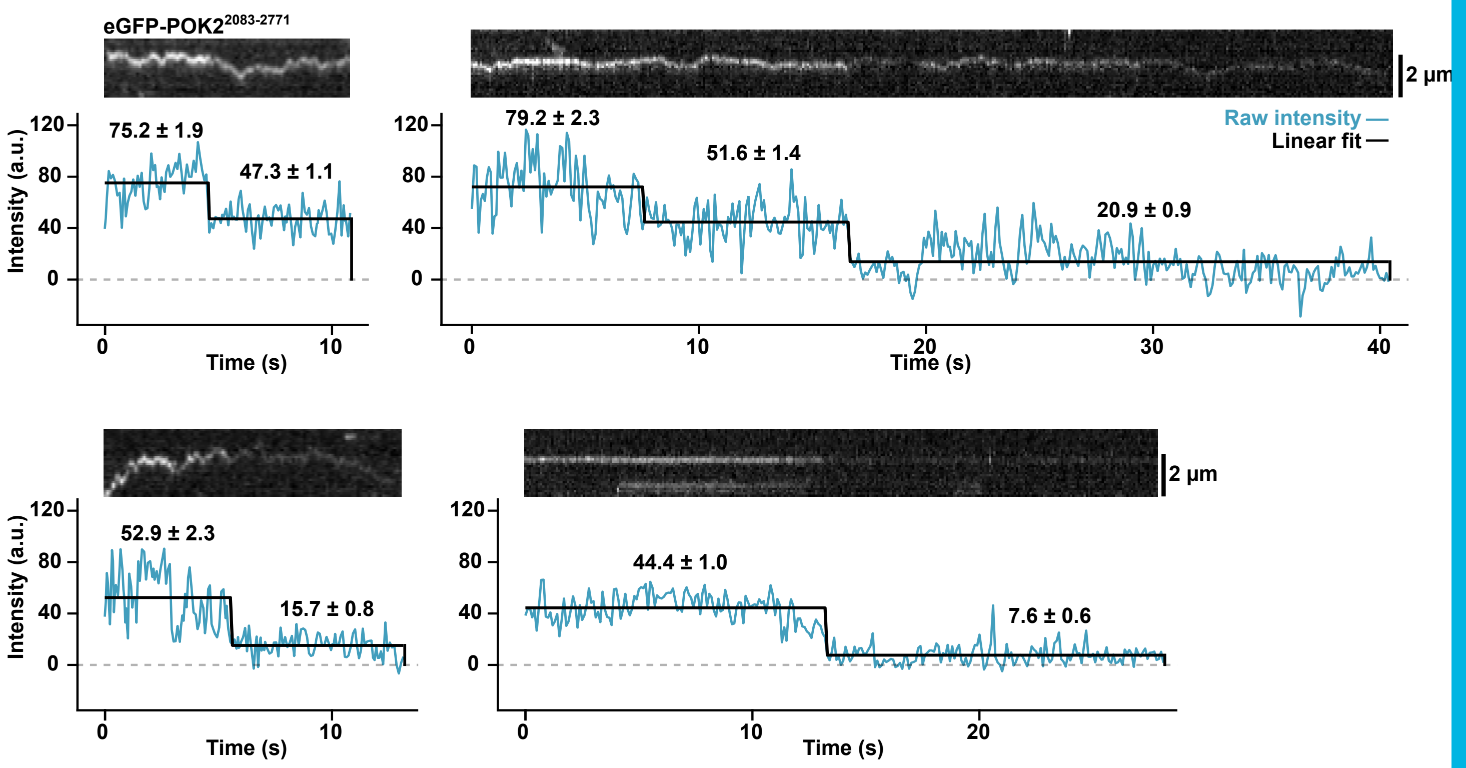

Fig. S2.

Background subtracted intensity in arbitrary units plotted against time for three diffusive and one stationary single molecule eGFP-POK2$^{2083-2771}$ traces on the microtubule. Images above graphs are corresponding kymographs of which intensities were measured from. Blue lines are the raw intensity, black lines are linear fits. Intensities ± s.e.m. of the linear fits are given.

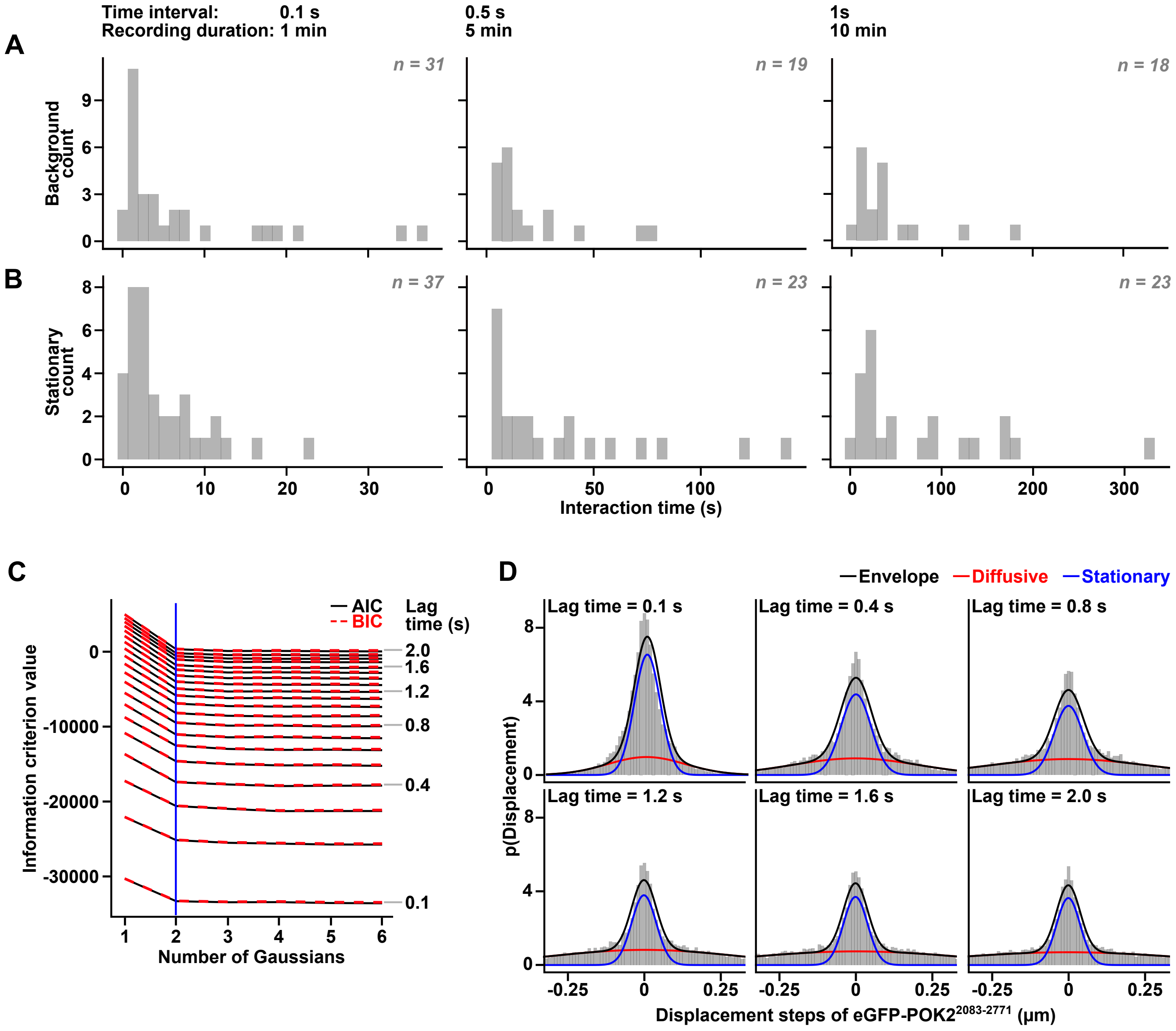

Fig. S3.

(A) Distribution of interaction times of events that were observed for kymographs from lines drawn in the background, where there were no microtubules present.

(B) Distribution of interaction times of stationary events that were observed for kymographs from lines drawn along microtubules.

(C) AIC and BIC were used to compare the relative quality of models for the displacement step distributions of eGFP-POK2$^{2083\text{-}2771}$ particles across lag times 0.1 to 2.0 s (sampled at 0.1 s intervals), fitted with one to six Gaussian components. The largest change in ICs were from one to two Gaussians (blue line), suggesting that two Gaussians are best fits for the displacement data.

(D) Displacement step distributions of eGFP-POK2$^{2083\text{-}2771}$ particles for representative lag times.

Journal of Cell Science • Supplementary information

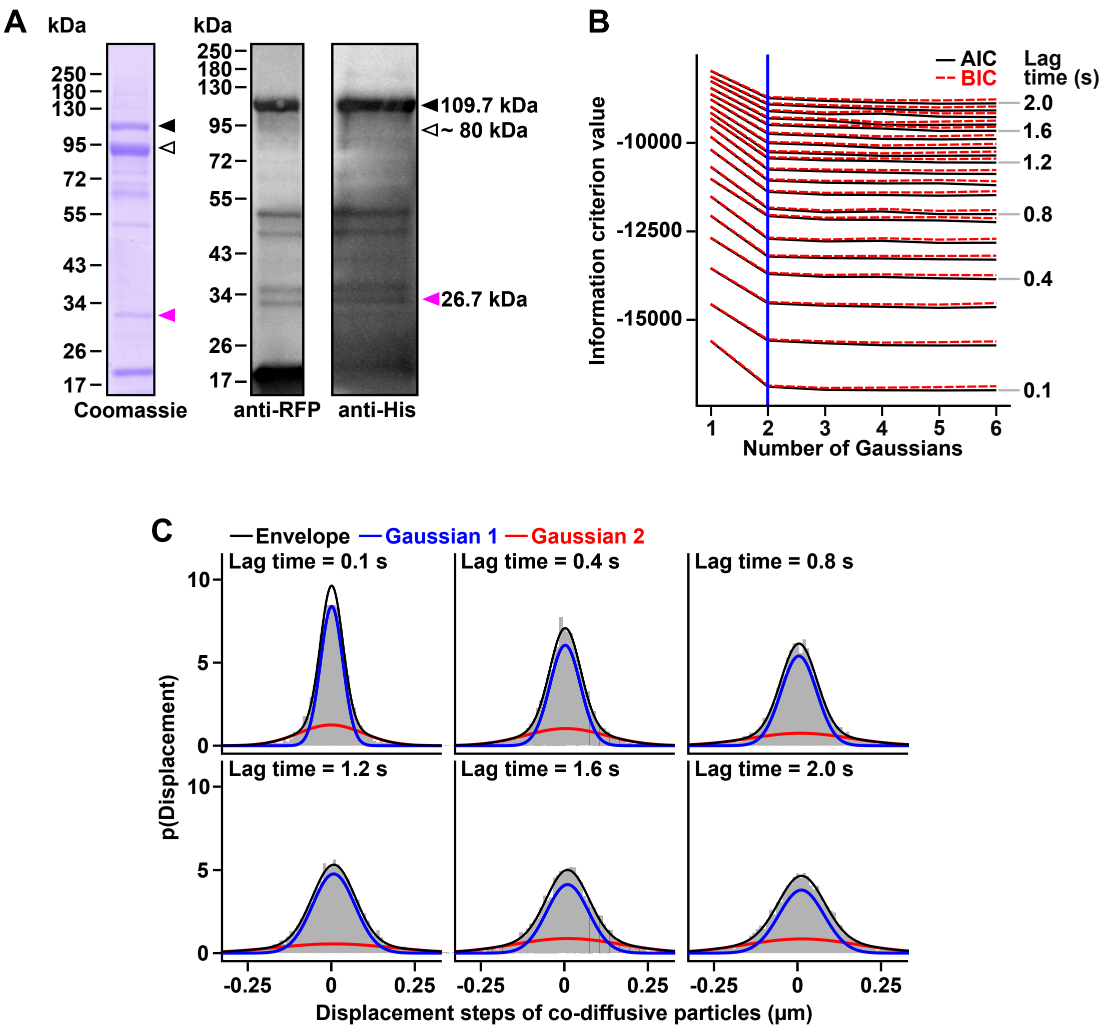

Fig. S4.

(A) Coomassie stained and western blots SDS-PAGE gel of MAP65-3-mCherry. Black arrow heads, predicted size of MAP65-3-mCherry. Empty arrow heads, predicted size of MAP65-3 without mCherry. Magenta arrow head, predicted size of mCherry fragment. Band pattern resembles that from previous publication (Ho et al., 2011, The Plant Cell).

(B) AIC and BIC were used to compare the relative quality of models for the displacement step distributions of co-diffusive MAP65-3-mCherry and eGFP-POK2$^{2083\text{-}2771}$ particles across lag times 0.1 to 2.0 s (sampled at 0.1 s intervals), fitted with one to six Gaussian components. Data indicated that the largest change in ICs to be from one to two Gaussians (blue line), suggesting that two Gaussians are best fits for the displacement data.

(C) Displacement step distributions of co-diffusive particles for representative lag times.

Journal of Cell Science • Supplementary information

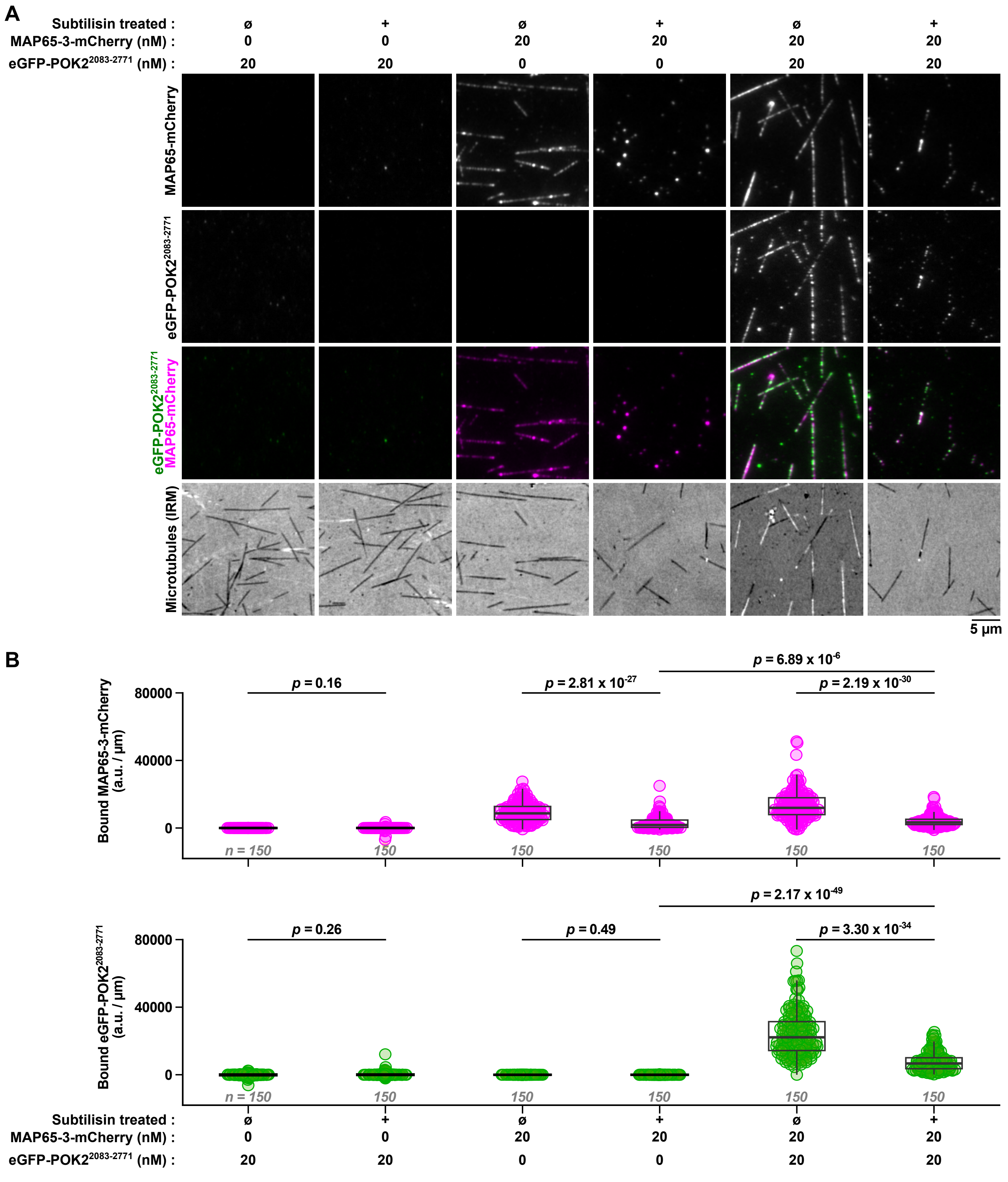

Fig. S5.

(A) TIRF images (*upper rows*, overlay in *third row*) and IRM images (*bottom row*) showing the binding of 20 nM eGFP-POK2$^{2083\text{-}2771}$ and/or 20 nM MAP65-3-mCherry to untreated (ø, *left columns*) or subtilisin-treated (+, *right columns*) double-stabilised biotinylated microtubules. Levels are the same across all TIRF images.

(B) Quantification of the microtubule binding of 20 nM MAP65-3-mCherry (*top*) and/or 20 nM eGFP-POK22083-2771 (*bottom*) from TIRF images such as in (A) in arbitrary intensity units per micrometer microtubule. *Data points* indicate single microtubules from three different flow chambers. *Horizontal bars* indicate the median, box is the interquartile range (IQR), whiskers are the Tukey range. *P-values* are derrived from a Mann-Whitney-Wilcoxon *U*-test.

Journal of Cell Science • Supplementary information

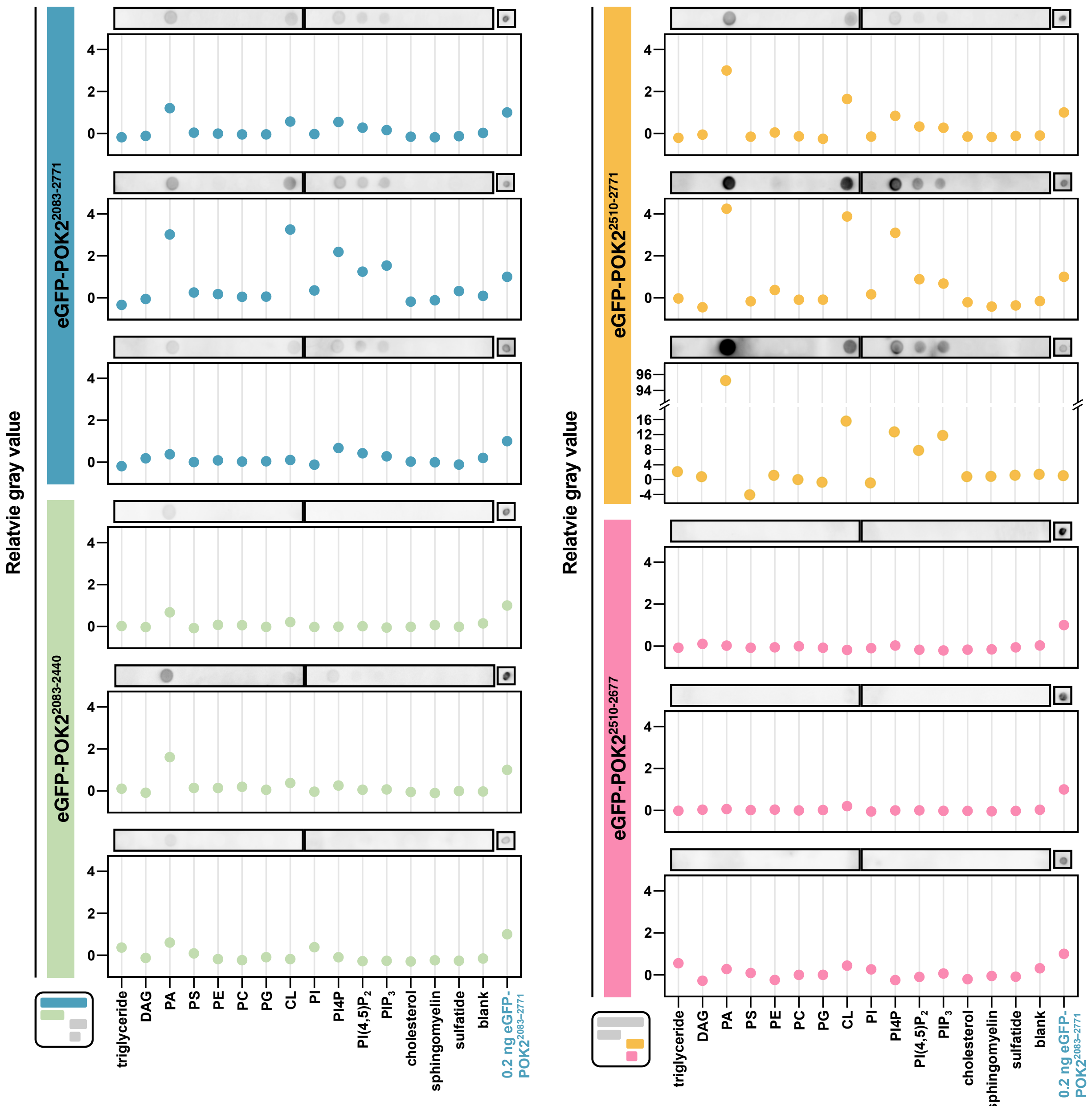

Fig. S6.

Individual lipid blot assays with indicated eGFP-POK2 fragments and immobilised lipids probed with anti-GFP antibodies corresponding to Fig. 7A. Images from lipid blots are shown above plots, which show quantification of signals normalised to loading control (i.e., 0.2 ng eGFP-POK22083-2771) directly dotted on the nitrocellulose membrane. Please note that brightness and contrast is individually adjusted for the images of the lipid blots.

Journal of Cell Science • Supplementary information

```
       - --- ++  +   -  -  -    + +   + -           -  -- +     +                 +-     +- 
1681 VYDMDEEVKR HRTTQDSLET ELQALRQRLF RFENFTGTMV TTNESTEEYK SHISRSTGLQ GAHSQIQVLQ KEVAEQTKEI 1760
     +  +-   -     -       -+ +  -    +- + --      -     + -+   + +       +          + -+
1761 KQLKEYISEI LLHSEAQSSA YQEKYKTLEV MIRDFKLEDS SSSAAETISH KTEKSSTRSR GSSSPFRCIV GLVQQMKLEK 1840
     - -    + +  -- -     + +-       +    -    -  +-    +  -     -   -     +  - +     --  
1841 DQELTMARVR VEELESLLAV KQKEICTLNT RIAAADSMTH DVIRDLLGVK MDITSYAELI DQHQVQRVVE KAQQHAEEIL 1920
      +- -    + +  -   +-+ -    -  ++ - -         -   -+         -  +  -+    ++   - -+      
1921 SKEQEVMNLK RHIDYLFKDR ESCMSELNKK DTDVLATQIS LDQLQERVQL LSMQNEMLKN DKSNLLRKLA ELDRTVHNAQ 2000
         +      +     +  -  -  ++ -    +       -   + ++         +         + +
2001 ASNHRVPQTT KDTASFKLAD TDYTKRLENA QKLLSHANNE LAKYRKTSNN HPSTRTQGQS SGTRYR                2066
```

Fig. S7. Amino acid sequence of the POK1 tail. The fragment comprising amino acids 1683- 2066 had been shown to interact with TAN , mediating long term retention of POK1 at the CDZ *(Muller at al. 2006, Lipka et al. 2014).* Amino acids with positively *(red)* and negatively *(light blue)* charged side chains are indicated.

Journal of Cell Science • Supplementary information

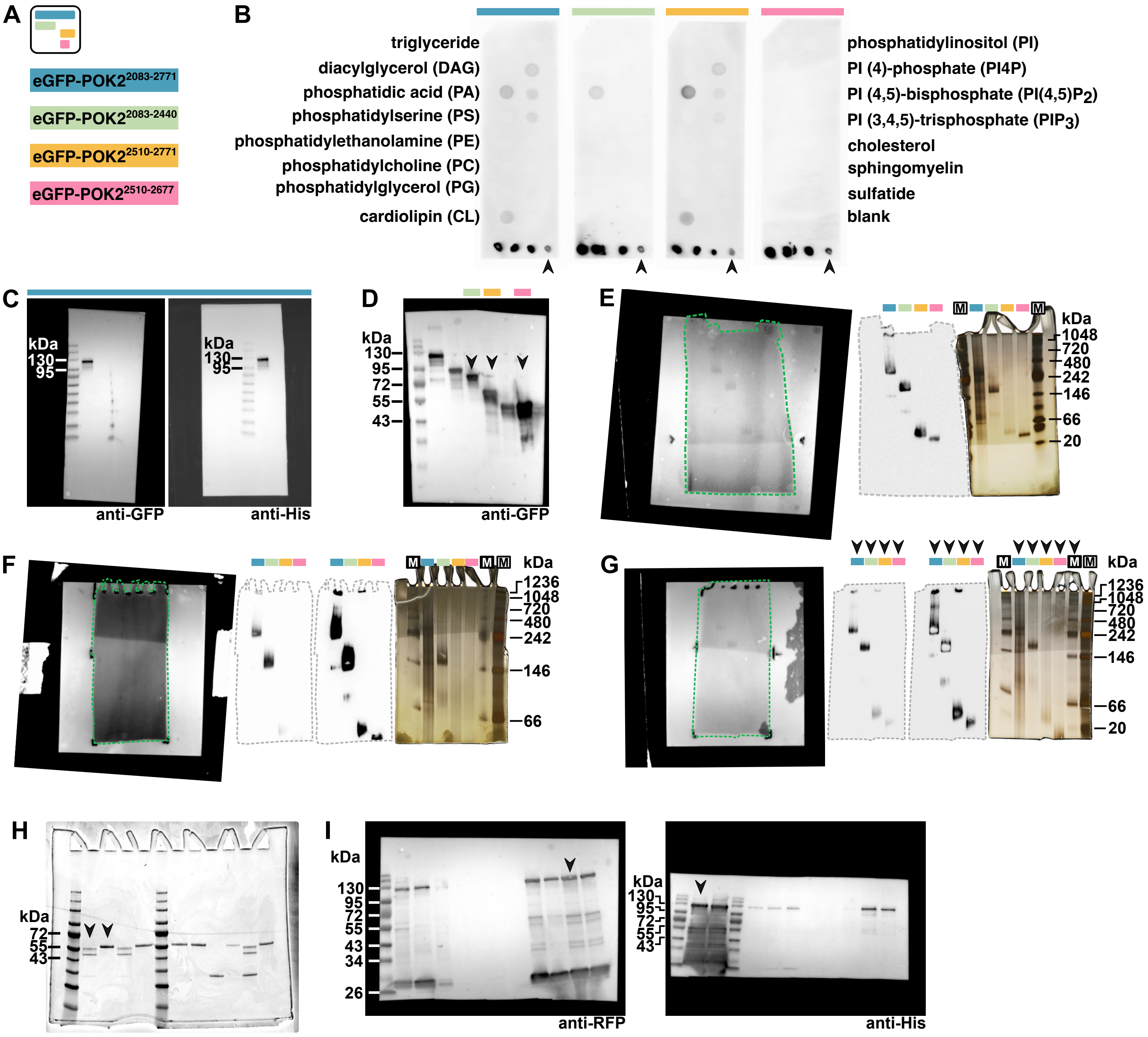

Fig. S8. Blot Transparency

(A) Square icons and colours representing eGFP-POK2 fragment used, arranged as in *Fig. 1A*. Colour code in (A–G) follows this icon.

(B) Uncropped images from lipid blot assay from *Fig. 7A*. Proteins were probed with anti-GFP (C7.1/13.1). 55, 20, 2 and 0.2 ng eGFP-POK2$^{2083\text{-}2771}$ (from left to right) were dotted at the bottom as controls. Black arrowhead indicates 0.2 ng eGFP-POK2$^{2083\text{-}2771}$ that was used to normalise signals for quantification in *Fig. 7A*.

(C) Uncropped images of anti-GFP and anti-His western blots of eGFP-POK2$^{2083\text{-}2771}$ from *Fig. S1A*.

(D) Uncropped images of anti-GFP western blot of shorter eGFP-POK2 tail fragments from *Fig. S1B*. Arrowheads indicate the lanes shown in *Fig. S1B.*

Journal of Cell Science • Supplementary information

(E–G) Uncropped images from anti-GFP western blots and silver stained Blue-Native PAGE gels of 100 ng eGFP-POK2 tail constructs as in *Fig. S1C*. On the left are uncropped images of the membrane taken with normal light, in order to mark position of the chemiluminescent bands relative to the blot position. This is done in order to compare it with the silver stained images, where the marker can then be visualised. Colors indicate eGFP-POK2 fragment used, colour coded as in (A). Black M in white box indicates marker, whereas white M in black box indicated 1:20 diluted marker. Green dashed outline corresponds to the grey dashed outlines. The blots and lanes shown in *Fig. S1C* are indicated with black arrowheads in (G).

(H) Uncropped image of Coomassie stained SDS-PAGE of subtilisin A treated and untreated microtubules from *Fig. S1D*. Lanes shown in *Fig. S1D* are indicated with black arrowheads.

(I) Uncropped images of anti-RFP and anti-His western blots of MAP65-3-mCherry from *Fig. S4A*. Arrowheads indicate lanes shown in *Fig. S4A*.

Table S1. Plasmids used in this research. Plasmids were made with the primers (Supplemental Table 2) using HiFi cloning (NEB) except S102 which was made by plasmid synthesis (GenScript).

ID-Nr.	Name	Resistance in *E. coli*
S59	eGFP-POK2$^{2083\text{-}2771}$-8xHis / pFastBac1	Ampicillin
S62	eGFP-POK2$^{2083\text{-}2440}$-8xHis / pFastBac1	Ampicillin
S64	eGFP-POK2$^{2510\text{-}2771}$-8xHis / pFastBac1	Ampicillin
S66	eGFP-POK2$^{2510\text{-}2677}$-8xHis / pFastBac1	Ampicillin
S102	MAP65-3-mCherry-8xHis / pFastBac1	Ampicillin

Table S2. Primers used in this research. Plasmid ID-Nr. refers to the listed plasmids in Table S1.

Name	Sequence (5'→3')	Used for cloning in plasmid ID-Nr.
Linker-POK2(2083) F	GACAAGAAAGATGAAACAAAGGAAATCATGGTTCATGTTG	S59, S62
Linker-POK2(2083) R	TTTGTTTCATCTTTCTTGTCGCCAGAACCAGCAGCGGAGCCAGCG	S59, S62
Linker-His F	GGAAGCGGATCTGGATCTGGACATC	S59, S62, S64, S66
POK2(2771)-linker-His R	CCAGATCCAGATCCGCTTCCCCTGTCTAAAGAAGAGAAAAAAGGAACCTG	S59, S64
POK2(2440)-linker-His R	CCAGATCCAGATCCGCTTCCCCTTGCAGCAGCAAGCTCCTCGTCC	S62
Linker-POK2(2510) F	GCTCCGCTGCTGGTTCTGGCGATTCACGAGAAAGGGATCTTGAAGTCTCT	S64, S66
Linker R	CGCTGGCTCCGCTGCTGGTTCTGGCGA	S64, S66
POK2(2677)-linker-His R	CCAGATCCAGATCCGCTTCCCTCCTGAGCAAGCTGCATTCTATCC	S66

Table S3. Survival plots of interaction times of diffusive eGFP-POK2$^{2083\text{-}2771}$ on microtubules were fitted with a sum of two exponentials (Refer Fig. 3E). The fitting equation is given as: $A_1 e^{-\left(\frac{x-t_0}{t_1}\right)} + A_2 e^{-\left(\frac{x-t_0}{t_2}\right)}$, where A is amplitude, t is time constants, with t_0 denoting the smallest time interval. Overall, three time constants, $t_{a,b,c}$, were obtained with two time constants for each recording time. Additionally in the table, t_{total} denotes recording time, Δt is the time interval, N is the number of traces, R^2 is the R-squared value of the fitting. Asterisk denotes fixed time constants during the fitting. Time constants and amplitude values are ± SE.

t_{total}	Δt	N	R^2	t_a	A_a	t_b	A_b	t_c	A_c
1 min	0.1 s	103	0.997	0.88 ± 0.05 s	0.58 ± 0.03	5.2 ± 0.4 s	0.40 ± 0.21		
5 min	0.5 s	73	0.990			6.0 ± 0.3 s	0.70 ± 0.02	*42.7 s	0.30 ± 0.02
10 min	1 s	74	0.996			*5.3 s	0.30 ± 0.02	42 ± 1 s	0.70 ± 0.01

Table S4. Estimation of frictional drag coefficient, γ and corresponding frictional force, $F_{friction}$. Diffusion coefficient is given ± SE. Frictional drag coefficient, γ was calculated using Einstein's relation, $\gamma = \frac{k_B T}{D}$, where k_B denotes Boltzmann's constant (Bormuth et al., 2009). Frictional force, $F_{friction}$ is calculated by multiplying γ by the velocity of the POK2 motor (0.43 ± 0.02 μm·s^{-1} (Chugh et al., 2018)).

	Diffusion coefficient, D (μm^{2}s^{-1})	Temperature, T (K)	Frictional drag coefficient, N·s·m^{-1})	Frictional force, $F_{friction}$ (pN)
Diffusive eGFP-POK2$^{2083\text{-}2771}$	0.031 ± 0.001	200	8.90 x 10^{-8}	0.038
Co-diffusive eGFP-POK2$^{2083\text{-}2771}$ and MAP65-3-mCherry	0.011 ± 0.001	203	2.55 x 10^{-7}	0.11
	0.0013 ± 0.0001	203	2.80 x 10^{-6}	1.20

Journal of Cell Science • Supplementary information

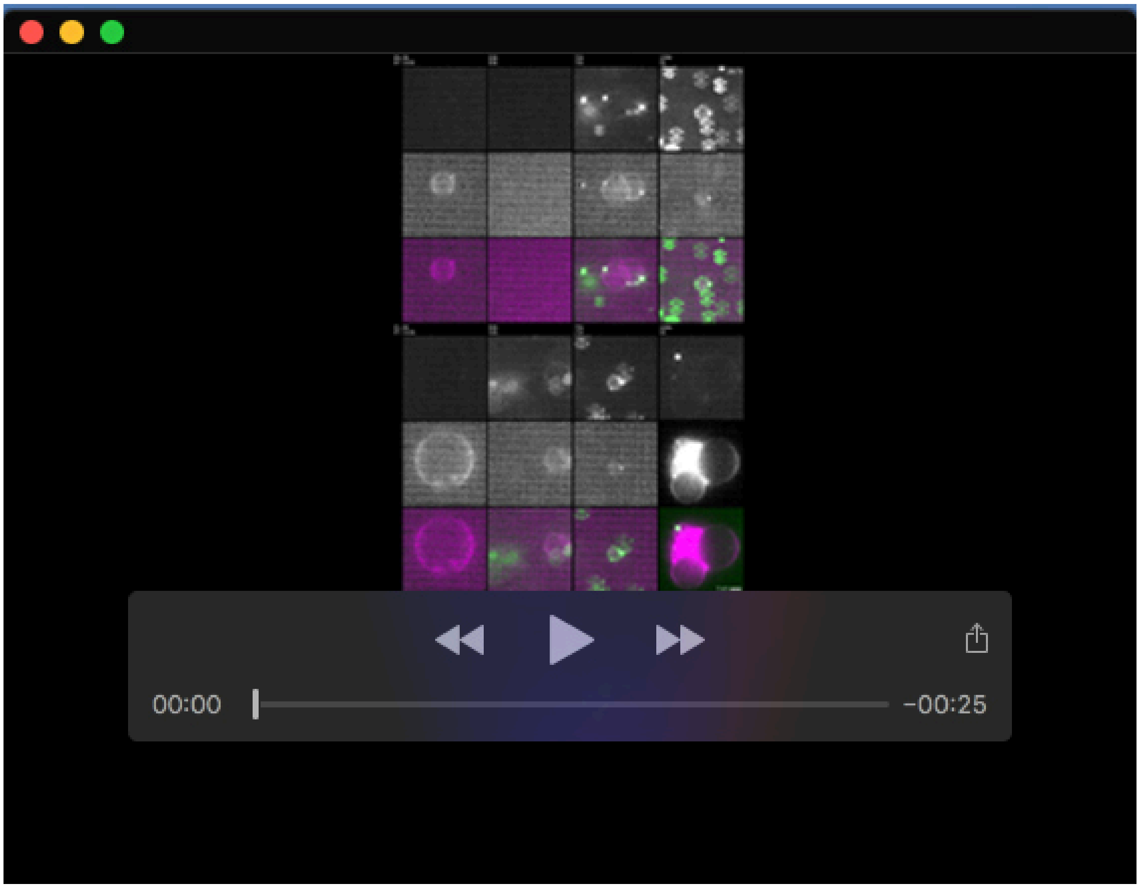

Movie 1. eGFP-POK2$^{2083\text{-}2771}$ binds to PA and CL GUVs.

Time lapse imaging series of eGFP-POK2$^{2083\text{-}2771}$ binding to giant unilamellar vesicles (GUVs) made from varying ratios of phosphatidic acid (PA) and cardiolipin (CL). GUVs were unlabelled and visualised with HILO microscopy when illuminated by reflections off fluorescent contaminations in the red channel. Images were sample every 100 ms for 30 s. Movies correspond to images shown in Fig. 7B.

Journal of Cell Science • Supplementary information
